# Psychometric properties of the social interaction anxiety scale and the social phobia scale in Hungarian adults and adolescents

**DOI:** 10.1186/s12888-021-03174-6

**Published:** 2021-03-26

**Authors:** Andras N. Zsido, Brigitta Varadi-Borbas, Nikolett Arato

**Affiliations:** 1 Institute of Psychology, University of Pécs, 6, Ifjusag street, Pécs, Baranya H-7624 Hungary; 2 Doctoral School of Psychology, ELTE Eötvös Loránd University, Budapest, Hungary; 3 Institute of Psychology, ELTE Eötvös Loránd University, Budapest, Hungary

**Keywords:** SIAS-6, SPS-6, Social anxiety disorder, Performance only specifier, Item response theory, Clinical sample, Adolescent validation

## Abstract

**Background:**

Although social anxiety disorder is one of the most frequent disorders, it often remained unrecognized. Utilizing brief, yet reliable screening tools, such as the Social Interaction Anxiety Scale (SIAS-6) and the Social Phobia Scale (SPS-6) are helping to solve this problem in parts of Western Europe and the US. Still some countries, like Hungary, lag behind. For this purpose, previous studies call for further evidence on the applicability of the scales in various populations and cultures, as well as the elaborative validity of the short forms. Here, we aimed to provide a thorough analysis of the scales in five studies. We employed item response theory (IRT) to explore the psychometric properties of the SIAS-6 and the SPS-6 in Hungarian adults (*n* = 3213, age range:19–80) and adolescents (*n* = 292, age range:14–18).

**Results:**

In both samples, IRT analyses demonstrated that the items of SIAS-6 and SPS-6 had high discriminative power and cover a wide range of the latent trait. Using various subsamples, we showed that (1) the scales had excellent convergent and divergent validity in relation to domains of anxiety, depression, and cognitive emotion regulation in both samples. Further, that (2) the scales discriminated those with a history of fainting or avoidance from those without such history. Lastly, (3) the questionnaires can discriminate people diagnosed with social anxiety disorder (*n* = 30, age range:13–71) and controls.

**Conclusions:**

These findings suggest that the questionnaires are suitable for screening for SAD in adults and adolescents. Although the confirmation of the two-factor structure may be indicative of the validity of the “performance only” specifier of SAD in DSM-V, the high correlation between the factors and the similar patter of convergent validity might indicate that it is not a discrete entity but rather a part of SAD; and that SAD is latently continuous.

**Supplementary Information:**

The online version contains supplementary material available at 10.1186/s12888-021-03174-6.

## Introduction

Social Anxiety Disorder (SAD) is one of the most frequent mental disorders with a lifetime prevalence of 5–13% [1–3]. People with SAD are afraid of and avoid certain social situations to such a degree that this causes significant impairment in their daily lives [4]. Possible feared situations include social interaction fears (e.g., initiating a conversation), fears about being observed (e.g., eating in front of other people), and performance fears (e.g., giving a speech). SAD, in general, can not only impair academic, career, and social functioning [5] but also dramatically decrease quality of life [6]. For example, people with SAD are more likely to drop out of school [7], be unemployed [8], and experience social isolation [9]. Around 90% of those who were diagnosed with SAD were also experiencing the symptoms of at least one other mental disorder [10], causing additional difficulties in their lives [5]. SAD most often precedes these comorbid disorders, especially depression and substance abuse [10, 11]. In addition, SAD can also predispose individuals to physical conditions like insomnia, diabetes, and autoimmune diseases [12, 13].

Although SAD is a greatly detrimental condition, it often remains unrecognized [14]. Even those who receive treatment generally find help only 15–20 years after the onset of their symptoms [2]. Consequently, it would be important to improve the detection of the disorder. For this reason, effective screening instruments that can be administered quickly, and are sensitive enough to correctly recognize those who could potentially be diagnosed with SAD would be necessary [15]. Brief, yet reliable instruments could not only serve the better recognition of the disorder but are also indispensable for measuring treatment progress in a more efficient way [16]. Furthermore, elderly populations and people with cognitive impairments might particularly benefit from shorter measures, as shorter measures could place less response burden on them [17]. Appropriate questionnaires are essential for these populations because anxiety is prevalent in elderly people and people with cognitive impairments [18, 19]. The six-item Social Interaction Anxiety Scale (SIAS-6) and Social Phobia Scale (SPS-6) [20] are the short versions of the original, 20-item SIAS and SPS questionnaires [21], and might be the most appropriate measures for screening in primary care for the symptoms of SAD [22].

We have selected the SIAS-6 and SPS-6 scales among other similar measures because they distinguish social interaction- from social performance anxiety which is important as although the “performance only” specifier has been included in DSM-V, only a handful of other questionnaires measure it directly [23]. Furthermore, these scales are very brief, widely used worldwide, and have been translated to other languages. An advantage of the SIAS and SPS, and thus SIAS-6 and SPS-6, is that they assess anxieties regarding social interactions and fears related to the scrutiny of others [20]. The difference between the scales is that the SIAS and SIAS-6 scales measure social interaction fears, for instance initiating a conversation. The SPS and SPS-6 scales measure fears about being observed and performance fears, for instance, eating in front of others, or giving a speech. Thus, together the two scales cover all feared situations with regard to social situations and, hence they are a good tool to screen social anxiety and SAD. In spite of this, the factor structure of the original SIAS and SPS was not clear for a long time (e.g., [24]). Thus, the development of the short scales was not only useful for reducing the number of items of the SIAS and SPS but also to uncover the factor structure of scales [25]. Peters and colleagues [20] suggested two separate, one-factor solution for SIAS-6 and SPS-6, which was later replicated [25]. However, a recent study that compared three different factor solutions for the questionnaires found only moderate support for the two-factor model [26]. Therefore, more data would be necessary to decide the best factor structure solution of the SIAS-6 and SPS-6, in clinical and healthy populations as well.

The debate whether the one or two factors solution is the best may have relevance to the matter of whether social performance anxiety is a part, a specifier, or qualitatively distinct type of SAD. An issue that has been long debated and has not yet been settled. Some claim that subtyping of SAD is necessary based on core fears. Although one of these, the “performance only” specifier has been introduced in DSM-V [4], recent meta-analyses on taxometric research showed that the “performance only” specifier introduced in DSM-V might not be a discrete entity but a part of SAD and that SAD is latently continuous [27, 28].

Both in terms of reliability and validity, the long version of the SIAS and SPS demonstrates adequate psychometric properties [21]. There are little data about the psychometric properties of the short versions, but the efficacy of the SIAS-6 and SPS-6 is still well supported [29]. Convergent validity was assessed by examining the correlations between the SIAS-6 and SPS-6, and questionnaires measuring fear of negative and positive evaluation, social anxiety symptoms, depression, worry, and anxiety sensitivity [20, 25, 30]. Diagnostic sensitivity was determined based on a receiver operating characteristic analysis (ROC), which analysis demonstrated the ability of the questionnaires to discriminate between people with and without SAD [20]. Sensitivity to treatment was assessed by comparing the scores on the questionnaires during the process of treatment [30, 31]. The internal consistency of the SIAS-6 and SPS-6 was .79 and .85 in the clinical, .75, and .82 in the nonanxious group [30]. These results indicate that the SIAS-6 and SPS-6 could be adequate for widespread use in clinical and research settings. Although the scales were already administered in various populations, for example, in samples with SAD, anxiety disorders, and university students [29, 30], previous studies call for further evidence on the applicability of the scales in various populations and cultures, as well as the elaborative validity of the short forms [25, 32].

There is a large body of previous evidence suggesting the role of emotional hyperreactivity in SAD [33, 34]. In fact, mindfulness-based and cognitive-behavioral therapies often seek to and capitalize on training emotion regulation skills [33–36]. While the failure of emotion regulation is thought to be a key feature of SAD [36–40], adaptive emotion regulation can reduce distress [36, 38]. In previous studies, convergent validity was only measured by examining the correlations between the SIAS-6 and SPS-6 scales and questionnaires measuring fear of negative and positive evaluation, social anxiety symptoms, depression, worry, and anxiety sensitivity [20, 25, 30]. In this study, our goal was to show that SIAS-6 and SPS-6 are associated with measures of adaptive and maladaptive emotion regulation skills to lend further support to the research and clinical applicability of the scales. Further, we sought to point to maladaptive and adaptive emotion regulation strategies that could either serve as risk or protective factors, respectively.

Social anxiety has a peak during the adolescent years [41–43] and numerous studies used SPS and SIAS in adolescent samples, the number of research examining the scale properties separately on an adolescent sample is scarce. We only found two studies to do so but they were either only using one of the scales [44] or used only a small sample size [29]. Thus, we sought to test whether the scales could also be used in this population, as there might be differences in the applicability of the questionnaires to various samples.

A previous study identified mismatches between the DSM criteria and the local phenomenology of SAD in specific cultural contexts [45]. Although there is mounting evidence [46–48] that the prevalence and expression of SAD are culture-dependent, most of the research on social anxiety has been conducted in the United States. Further, the SIAS and SPS scales have only been used in Australian, American, Japanese, and South Korean samples [29, 49]. This is a considerable limitation, as SAD has been implicated in avoidance of psychological services for individuals from different backgrounds and nationalities [50–52]. SAD may take different forms depending upon cultural norms [45, 47, 52]. We did not expect any cultural disparity for SAD that is particular to Hungary, then this study is mainly testing a translation of the scale. Thus, in this study we mainly sought to examine the psychometrical properties of SIAS-6 and SPS-6 in a slightly different, Central-European culture and language (i.e. Hungarian); and to lend further support to the wide applicability of the SIAS-6 and SPS-6 scales.

The current study aimed to gain more information about the factor structure and psychometric properties of the SIAS-6 and SPS-6 questionnaires in the Hungarian population. Although previous studies showed the clinical utility of the scales [29, 30], this has only been done on American samples. Since the prevalence and expression of SAD are culture-dependent [46–48], examining the clinical applicability of SIAS-6 and SPS-6 in other cultures is necessary. As social anxiety often develops during adolescence [53], apart from an adult and a clinical sample, we also recruited an adolescent community sample to evaluate whether the questionnaires are suitable for screening for SAD in adolescents. Specifically, besides examining the factor structure, we investigated the reliability of the scales by conducting item-response analyses and by examining item-total correlations and internal consistency values. In terms of validity, convergent, divergent, and predictive validity, as well as clinical specificity was examined.

## Method

### Participants

We used four separate samples in this study. The first sample comprised 3213 Hungarian participants. They ranged in age from 19 to 80 years (M = 29.4, SD = 12.1) and were predominantly female (71.5%). Our goal was to obtain a heterogeneous sample representing people from a variety of demographic, socio-economic, and educational backgrounds. We recruited participants throughout the Internet by posting recruitment notices in Hungarian to various frequently visited forums and several University mailing lists. Participants were also encouraged to help share the survey with their friends, family, and acquaintances. There were no eligibility restrictions to participate in the study. All respondents filled out the questionnaires online, using Google Forms.

The second sample comprised of 292 Hungarian adolescents participated. They ranged in age from 14 to 18 years (M = 17.6, SD = .87) and were predominantly females (72.3%). The incidental sample of adolescents came from several secondary education schools across Hungary. After obtaining consent from the teachers and the parents of the youths, the students were assessed. The self-reports were completed online using Google Forms but collectively in the classroom. Participants in the first and second samples filled out the scales as part of various other, larger studies. The time to fill out these studies was approximately 30–45 min.

The third sample comprised 63 undergraduate students (M = 22.1, SD = 1.43, 38 females) to assess the three-week test-retest reliability of the SIAS-6 and SPS-6 scales. All respondents filled out the questionnaires online, using Google Forms. None of the participants in the first three samples reported having clinically diagnosed SAD.

The fourth sample was a clinical sample and consisted of 30 participants, all out-patients of the local psychiatry clinic where they received their diagnosis based on clinical interviews conducted by psychiatrists or clinical psychologists. There were 13 adolescents (age range: 13–18, M = 15.2, SD = 1.69) and 17 adults (age range: 19–71, M = 44.6, SD = 20.39), the participants were predominantly female (69.2 and 70.6%; respectively). All individuals in this sample had a secondary diagnosis of social phobia, their primary diagnosis was either emotionally unstable personality disorder or mixed anxiety and depressive disorder. The clinical sample was obtained at an outpatient psychiatric clinic of the University. A clinical psychologist working at the clinic asked individuals upon arrival to the clinic whether they would fill out a short survey to help us validate a questionnaire. If they agreed, a self-report questionnaire was given to them, which they were requested to fill in at home and bring along to their next visit. A matching control sample based on age and gender was obtained by randomly selecting participants from the adult and adolescent samples. Participants in the third and fourth samples were directly recruited for this study. The time to fill out the test battery was approximately 5 min.

We used three subsamples of adults (first sample) and adolescents (second sample) to access convergent, divergent, and predictive validity. The first subsample completed other questionnaires to access convergent and divergent validity. This subsample comprised of 210 participants, ranging in age from 15 to 68 years (M = 34.8, SD = 13.4) and were predominantly females (84.3%). The second subsample was also used to access convergent validity with a different questionnaire than in the first subsample. This subsample comprised 410 participants ranging in age from 15 to 75 years (M = 32.2, SD = 12.9) and were predominantly females (80.2%). A third subsample was used to access predictive validity. This subsample comprised 743 participants who ranged in age from 15 to 75 years (M = 31.1, SD = 13.3) and were predominantly females (78.1%).

There were no missing data because, for those who completed the survey online, the answer was made mandatory for each question in the surveys. As for the clinical sample, we emphasized not to miss the answer to any questions in the instruction of the survey. We did not find any indicators of bot responses, and we did not expect to see any because participants completed all surveys voluntarily and in no instance were given any compensation. We sought for outliers who were ± 3 SDs away from the mean but we found non (which is justified by the large sample size). We also sought duplicate responses and identified seven in the first sample, these were removed and not analyzed or mentioned in the sample description.

### Compliance with ethical standards

All studies presented in this paper were approved by the Hungarian United Ethical Review Committee for Research in Psychology (nr. 2018–25) and were carried out in accordance with the Code of Ethics of the World Medical Association (Declaration of Helsinki). Written informed consent was obtained from all participants or their parents if they were under the age of 18. All the participants were given the same instructions to answer, and participation was voluntary.

### Questionnaires

We used the short forms of the SIAS and SPS scales, i.e. *SIAS-6 and SPS-6* [20]. The SIAS-6 is a self-report measure consisting of 6 items, intended to measure general anxiety associated with the initiation and maintenance of social interactions. The SPS-6 is also a self-report measure consisting of 6 items, intended to measure the experience of anxiety associated with the performance of various tasks while being scrutinized by others. Items are rated on a 5-point Likert-type scale with values ranging from 0 “Not at all characteristic or true of me” to 4 “Extremely characteristic or true of me”. The authors developing the short forms suggested a two-factor model for the companion scales because they were designed to measure two related facets of social anxiety [20, 21]. All of the participants filled out the Hungarian language versions of the scales.^1^ The process of translation and adaptation of the instruments followed the recommendations of the American Psychiatric Association [4]. First, the original version of the questionnaire was given to two psychologists, both of whom were fluent in English, to translate the SIAS-6 and SPS-6 scales to Hungarian. Then, a third person, an expert in test development, was asked to compare the two versions and merge them into one to avoid any discrepancies and mistranslations. Subsequently, a person with a Master’s degree in psychology who is fluent in English translated this version back to English. Thereafter, an expert panel consisting of researchers in psychology as well as a native English speaker reviewed the back-translated version. They revised and corrected the Hungarian version to make it as close as possible in meaning to the original SIAS-6 and SPS-6 scales. Since there are no cultural disparities for social anxiety disorder that are particular to Hungary, we did not change any aspect of the original scales. The only difference between the English and the Hungarian version is that in SIAS item 2 we appended the noun classmates (i.e., “I find it difficult mixing comfortably with the people I work with *or my classmates*.”) to make it more suitable for testing adolescents.

The *trait scale of the Spielberger State-Trait Anxiety Inventory* (STAI) was used to measure anxiety symptom severity [54]. Participants rated each item on a 4-point Likert-type scale ranging from “Not at all” to “Very much so”. The STAI demonstrates adequate reliability and validity; in this study, the McDonald’s ω was .91.

We administered the *Beck Depression Inventory* (BDI) [55] to measure depressive mood. Items were presented on 4-point scales. The BDI has adequate psychometric properties, in our study the McDonald’s ω was .87.

We used the 8-item brief version of the *Fear of Negative Evaluation* (bFNE) questionnaire [56]. All items are rated on a 5-point Likert-type scale ranging from 1 “Not at all characteristic of me” to 5 “Entirely characteristic of me” with higher scores implying higher fear of negative evaluation by others. The questionnaire has excellent psychometric properties, in our study the McDonald’s ω was .95.

We used the abbreviated, 12-item version of the *Snake Questionnaire* (SNAQ) [57] to measure fear of snakes. Participants answered the questions using a dichotomous response format (true; false). The SNAQ has been shown to have excellent psychometric properties, the McDonald’s ω was .92 in this study.

We used the 36-item version of the *Cognitive Emotion Regulation Questionnaire* (CERQ) [58]. The questionnaire measures a total of nine adaptive (Putting into Perspective, Positive Refocusing, Positive Reappraisal, Acceptance, and Planning.) and maladaptive emotion regulation strategies (Self-blame, Other-blame, Rumination, and Catastrophizing). Items are measured on a 5-point Likert-type scale ranging from “almost never” to “almost always”. The psychometric properties of the CERQ-short have been proven to be good, in our study McDonald’s ω values ranged from .60 to .78.

A subsample also answered two additional yes/no *questions that were based on DSM-V criteria* (historical instances of fainting and avoidance) for specific phobias [59]. Fainting and dizziness were assessed by asking: “Have you ever been so scared in a situation where you had to interact with others or others watched you that you fainted, nearly fainted, and felt dizzy or were not able to move?”. Avoidance was assessed by asking: “Have you ever avoided certain situations/places or procrastinated about different activities because you thought you might have to interact with others or others will watch you?”

### Statistical analyses

#### Confirmatory factor analyses (CFA)

One of the assumptions of the Item response theory (IRT) model we planned to use is the unidimensionality of the scales, i.e. all items load sufficiently onto one underlying construct. Thus, before conducting the item response analysis, we tested the two-factor model suggested by previous studies with confirmatory factor analysis. We used the diagonally weighted least squares (DWLS) estimator. To assess model fit, we used the comparative fit index (CFI), the Tucker–Lewis index (TLI), the root mean square error of approximation (RMSEA), and the standardized root mean squared residual index (SRMR). The cutoffs for good model fit were CFI and TLI values of .95 or greater [60], RMSEA and SRMR values of .08 or lower [61]. McDonald’s omega values were also calculated to access the reliability of the scales.

#### Examining the test characteristics of SIAS-6 and SPS-6

IRT models originally were utilized in educational testing, however, are now being implemented in health status assessment. In general, IRT is focused on the psychometric properties of the items and can show how informative each item is regarding the measured latent trait [62]. Traditional techniques cannot provide such information [63]. Moreover, IRT can useful in the calibration of tests in new languages [64]. Regarding the measurement of mental disorders, IRT can evaluate whether certain items are informative only at extreme severity levels of pathology or if they are useful for scaling severity across a wide range of pathology. That is, if the greatest utility of a scale is in differentiating participants close to one standard deviation above the mean in the distribution, that means the test can identify those with more severe problems than the average. Further, IRT allows for the examination of specific properties of each item of a scale to test which item significantly indicate a probable psychopathology, and to identify the level of severity of the problem at which the items are most informative [65].

Our primary goal was to conduct an item response analysis using the unidimensional graded response model (GRM). This model specifies a discrimination parameter (a) and a difficulty parameter (b) for each item. The a parameter shows how strongly the item is related to the latent variable, and the b parameter indicates where on the latent continuum the discrimination occurs [62]. We also calculated the item information curves (IICs) for all items, as well as test information functions (TIF) for both scales.

#### Accessing the validity of the scales

On the first subsample, we used correlational analyses between the questionnaires to assess the convergent (with STAI, BDI, and bFNE) and divergent (with SNAQ) validity of the SIAS-6 and SPS-6 scales. We used Spearman correlation because the SPS-6 (Skewness = 1.58, Kurtosis = 2.13) and BDI (Skewness = 1.75, Kurtosis = 3.13) had a slight negative binomial distribution. Then, on the second subsample, we used linear regression analyses (enter method) to explore which adaptive and maladaptive emotion regulation strategies could predict SIAS-6 and SPS-6 scores. In the models, the SIAS-6 and SPS-6 scores served as the dependent variables, while the nine subscales of CERQ were the independent variables. The Durbin-Watson tests were nonsignificant (DW = 1.88, *p* = .20 for SIAS-6 and DW = 1.96, *p* = .69 for SPS-6), VIF values were smaller than 1.7, Cook’s distances were smaller than .07 and the residuals were normally distributed.

To access predictive validity on the third subsample, we conducted a one-way analysis of variance (ANOVA) to compare the four groups on the SIAS-6 and SPS-6 total scores: fainting history only, avoidance history only, fainting and avoidance history, neither fainting history nor avoidance history. Then, we conducted a discriminant analysis to predict whether SIAS and SPS total scores can predict self-reported fainting and avoidance history.

#### Clinical specificity

We used Pearson’s correlations to observe the relationship between test scores and age. Finally, we calculated group differences for the clinical and non-clinical samples using independent samples t-tests on all scales.

## Results

### Confirmatory factor analysis

On the *adult sample,* the two-factor CFA yielded an adequate level of fit on our sample (CFI = .978, TLI = .972, RMSEA = .051, 90%CI = [.047–.056], SRMR = .025).^2^ Factor loadings varied between .53 and .82 on the SIAS-6 scale and between .67 and .82 on the SPS-6 scale. Further, both the SIAS-6 and SPS-6 scales demonstrated good internal consistency (McDonald’s ω was .87 for the SIAS-6 and .88 for the SPS-6) and corrected item-total correlations (range: .48–.76 for the SIAS-6 and .63–.76 for the SPS-6).

The three-week test-retest reliability on an independent sample for the total scores of both questionnaires proved to be high, with Pearson correlations (r) of .90 for the SIAS-6 and .92 for the SPS-6.

On the *adolescent sample,* the two-factor CFA yielded an adequate level of fit on our sample (CFI = .970, TLI = .962, RMSEA = .049, 90%CI = [.032–.067], SRMR = .035). Factor loadings varied between .45 and .74 on the SIAS-6 scale and between .59 and .79 on the SPS-6 scale. Further, both the SIAS-6 and SPS-6 scales demonstrated good internal consistency (McDonald’s ω was .82 for the SIAS-6 and .83 for the SPS-6) and corrected item-total correlations (range: .40–.68 for the SIAS-6 and .54–.68 for the SPS-6).

Regarding the *clinical sample,* both the SIAS-6 (McDonald’s ω = .87) and SPS-6 (McDonald’s ω = .88) total scores, as well as bFNE total score (McDonald’s ω = .92), demonstrated good internal consistency in this sample.

*Examining the test characteristics of SIAS-6 and SPS-6.*

Regarding the *adult sample,* as the CFA confirmed that both scales had a single latent variable, the scales were analyzed separately using GRM IRT. The a values ranged between 1.11 to 3.44 for the SIAS-6 scale and between 2.03 to 3.12 for the SPS-6 scale showing that the items of the two scales had very high discrimination values based on the recommended threshold of 1.7 [62]. The only exception was item 6 of SIAS-6 with a = 1.11 which can be considered moderate discrimination power. Therefore, the scales can discriminate on a wide range of social anxiety levels represented by the underlying latent variable. Table 1 shows the a parameter values for each item.

**Table 1:** The discrimination parameters (a) for each item of the Social Interaction Anxiety Scale (SIAS-6) and the Social Phobia Scale (SPS-6) in the total adult community sample (*N* = 3213) and the adolescent community sample (*N* = 292)

Questionnaire	Item number	Adult sample	Adolescent sample
		*a* value (std error)	
SIAS-6	1	1.98 (.07)	1.53 (.19)
	2	2.86 (.10)	1.82 (.22)
	3	2.35 (.08)	1.95 (.25)
	4	2.63 (.09)	1.98 (.24)
	5	3.44 (.13)	2.54 (.33)
	6	1.11 (.05)	.87 (.16)
SPS-6	1	2.34 (.08)	1.65 (.21)
	2	1.98 (.07)	1.43 (.19)
	3	2.51 (.09)	1.74 (.22)
	4	2.91 (.11)	2.33 (.29)
	5	2.79 (.10)	2.57 (.33)
	6	2.03 (.08)	1.47 (.19)

**Fig. 1:**
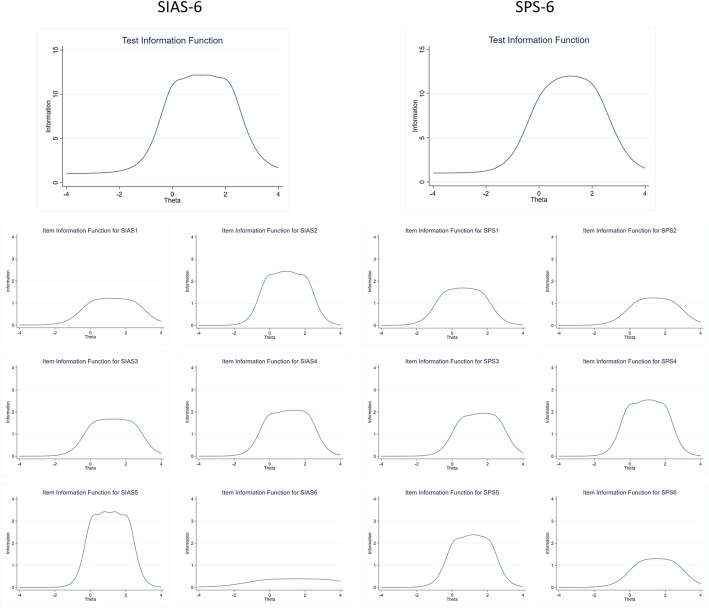
Test Information Function and Item Information Curves on the adult sample for both the Social Interaction Anxiety Scale (SIAS-6; right) and the Social Phobia Scale (SPS-6; left)

For both scales, the IICs demonstrated that items provided the most information about the latent ability for different ability levels. Figure 1 shows a plot of the psychometric information each item contains over the range of the latent variable. The TIFs demonstrated good coverage of a wide range of latent ability levels, see Fig. 1. Overall, the mean of ability scores is around 1.5, and the standard deviation is about 1. The tests had more information on people who were more prone to social anxiety. The TIF curves are approximately normal-shaped.

On the *adolescent sample*, as the CFA confirmed that both scales had a single latent variable, the scales were analyzed separately using GRM IRT. The a values ranged between .87 to 2.54 for the SIAS-6 scale and between 1.43 to 2.57 for the SPS-6 scale showing that the items of the two scales had mostly high discrimination values based on the recommended threshold of 1.7 [62]. Regarding the SIAS-6 scale, one item had moderate, one item had high and four items had very high discrimination ability. For the SPS-6, four out of six items had high, while two items had very high discrimination ability. Thus, the scales can discriminate on a wide range of social anxiety levels represented by the underlying latent variable. Table 1 shows the a parameter values for each item.

**Fig. 2:**
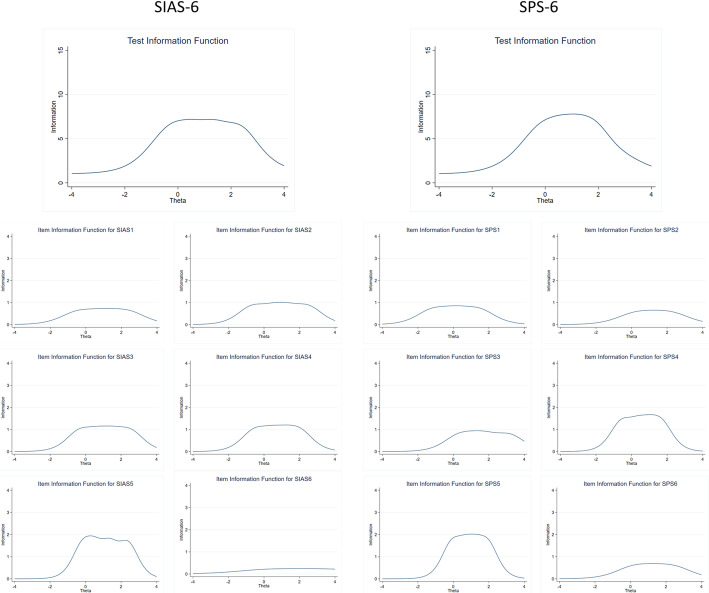
Test Information Function and Item Information Curves on the adolescent sample for both the Social Interaction Anxiety Scale (SIAS-6; right) and the Social Phobia Scale (SPS-6; left)

For both scales, the IICs showed that the various items provided the most information about the latent ability for different ability levels. Figure 2 shows a plot of the psychometric information each item contains over the range of the latent variable. The TIFs demonstrated good coverage of a wide range of latent ability levels, see Fig. 2. Overall, the mean of ability scores is around 1, and the standard deviation is about 1. The tests have more information on people who are more prone to social anxiety. The TIF curves are approximately normal-shaped.

### Demographic analysis

On the *adult sample,* both SIAS-6 and SPS-6 total scores correlated negatively with age (r = −.247, *p* < .001 and r = −.246, p < .001; respectively) and were significantly higher among females than among males (t(3208) = 4.88, p < .001, Cohen’s d = .19 and t(3207) = 8.03, p < .001, Cohen’s d = .31; respectively). Mean scores and standard deviations are displayed in Table 2. Mean scores on all items ranged between .54 and .1.34 suggesting that the content of most social anxiety items was generally outside of the experience of most participants.

**Table 2:** Descriptive statistics, internal consistency (McDonald’s ω) and fit statistics for the two-factor model for the total adult community sample (Study 1), the adolescent community sample (Study 4), and the clinical sample (Study 5) on the six-item versions of the Social Interaction Anxiety Scale (SIAS-6) and Social Phobia Scale (SPS-6)

		Study 1 - Total adult community sample	Study 4 - Community adolescent sample	Study 5 - Clinical sample
	N	3213	292	30
Questionnaire				
SIAS	M (SD)	5.41 (5.26)	6.63 (5.09)	10.67 (5.10)
	Females: M (SD)	5.69 (5.37)	6.92 (5.32)	10.65 (4.91)
	Males: M (SD)	4.69 (4.89)	5.88 (4.36)	11.67 (5.15)
	McDonald’s ω	.87	.82	.87
SPS	M (SD)	5.00 (5.37)	6.74 (5.49)	10.27 (5.86)
	Females: M (SD)	5.47 (5.55)	7.26 (5.56)	10.45 (6.41)
	Males: M (SD)	3.80 (4.69)	5.37 (5.06)	10.89 (3.95)
	McDonald’s ω	.88	.83	.92
Fit statistics		CFI = .978, TLI = .972, RMSEA = .051 (90%CI = .047–.056), SRMR = .025	CFI = .970, TLI = .962, RMSEA = .049 (90%CI = .032–.067), SRMR = .035	

*N* Number of participants, *M* Mean, *SD* Standard Deviation

Regarding the *adolescent sample,* neither SIAS-6 nor SPS-6 total scores correlated with age (r = .039 and r = .002; respectively). Although females scored higher than males on both scales, this difference was not significant on the SIAS-6 scale (t(290) = 1.58, *p* = .115, Cohen’s d = .21), only on the SPS-6 scale (t(290) = 2.66, *p* = .008, Cohen’s d = .35). Mean scores and standard deviations are displayed in Table 2. Mean scores on all items ranged between .71 and 1.32 suggesting that the content of most social anxiety items was generally outside of the experience of most participants.

On the *clinical sample*, the total scores showed week to moderate negative correlations with age (SIAS-6: r = −.24, *p* = .07; SPS-6: r = −.27, *p* = .04; bFNE: r = −.31, *p* = .02).

### The validity of SIAS-6 and SPS-6

Regarding *convergent and divergent validity*, the SIAS and SPS values correlated strongly. Both the SIAS-6 and SPS-6 showed medium to strong positive correlations with the STAI, BDI, and bFNE scales and a nonsignificant correlation with SNAQ. See Table 3 for the correlational coefficients.

**Table 3:** Spearman correlational coefficients (rho) on a subsample (*N* = 210) between the Social Interaction Anxiety Scale (SIAS-6) and the Social Phobia Scale (SPS-6) and the Spielberger Trait Anxiety Inventory (STAI), the Beck Depression Inventory (BDI), the Brief Fear of Negative Evaluation scale (bFNE) and the Snake Questionnaire (SNAQ)

	STAI	BDI	bFNE	SNAQ	SPS
SIAS-6	.478**	.391**	.627**	.002	.660**
SPS-6	.474**	.391**	.636**	.021	–

** < .001, * < 0.01

Regarding the SIAS-6 total score, the linear regression model (F(9,400) = 10.25,*p* < .001, adjusted R^2^ = .17) showed that positive reappraisal negatively predicted the scores on the questionnaire (β = −.15, 95%CI: −.70 to −.10, *p* = .008). Whereas, the factors that positively predicted the score were self-blame (β = .20, 95%CI: .28 to .83, p < .001), acceptance (β = .14, 95%CI: .09 to .69, *p* = .011), and rumination (β = .23, 95%CI: .28 to .84, p < .001). See Table 4 for the exact values.

**Table 4:** Detailed results of the linear regressions on a subsample (*N* = 410) separately for the Social Interaction Anxiety Scale (SIAS-6) and the Social Phobia Scale (SPS-6) with the nine Cognitive Emotion Regulation Questionnaire subscales as predictors. The table shows the point estimates (B), standard errors (SE), standardized estimates (β), 95% confidence intervals (95%CI) for the standardized estimates

	SIAS-6							SPS-6						
**Variables**	**B**	**SE**	**β**	**t**	**p**	**95%CI**		**B**	**SE**	**β**	**t**	**p**	**95%CI**	
						**lower**	**upper**						**lower**	**upper**
Self-blame	.55	.14	.20	3.93	<.001	.28	.83	.39	.14	.14	2.82	.005	.12	.67
Acceptance	.39	.15	.14	2.55	.011	.09	.69	.13	.15	.05	.86	.388	−.17	.43
Rumination	.56	.14	.23	3.94	<.001	.28	.84	.69	.14	.28	4.83	<.001	.41	.97
Positive refocusing	−.14	.14	−.06	−1.01	.315	−.40	.13	−.21	.14	−.08	−1.52	.130	−.47	.06
Planning	−.04	.18	−.01	−.24	.813	−.40	.31	−.16	.18	−.05	−.86	.388	−.51	.20
Positive reappraisal	−.40	.15	−.15	−2.65	.008	−.70	−.10	−.17	.15	−.06	−1.10	.273	−.46	.13
Perspective	−.14	.16	−.05	−.86	.390	−.45	.18	−.01	.16	.00	−.07	.947	−.32	.30
Catastro-phizing	.16	.16	.06	.98	.328	−.16	.48	.40	.16	.14	2.46	.014	.08	.72
Other-blame	.04	.18	.01	.22	.825	−.31	.39	−.11	.18	−.03	−.62	.537	−.46	.24

Regarding the SPS-6 total score, the linear regression model (F(9,400) = 10.33,*p* < .001, adjusted R^2^ = .17) showed that self-blame (β = .14, 95%CI: .12 to .67, *p* = .005), rumination (β = .28, 95%CI: .41 to .97, p < .001), and catastrophizing (β = .14, 95%CI: .08 to .72, *p* = .014) positively predicted the score. See Table 4 for the exact values.

Regarding *predictive validity*, a total of 126 participants reported a history of fainting/dizziness alone, 31 participants reported a history of avoidance alone, 223 reported a history of both fainting/dizziness and avoidance, and 363 participants reported no history of either fainting/dizziness or avoidance.

**Fig. 3:**
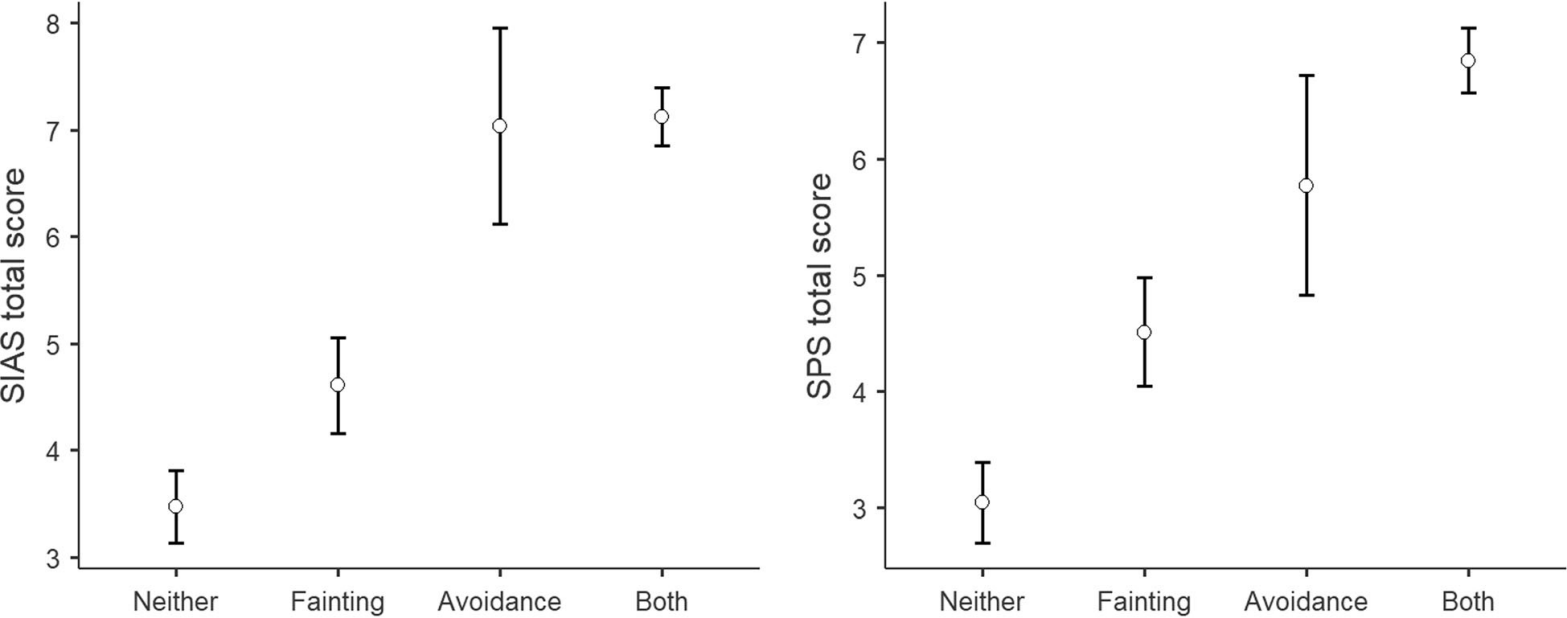
Group differences for those with neither a fainting history nor an avoidance history, a fainting history only, avoidance history only, both fainting and avoidance history, and on the Social Interaction Anxiety Scale (SIAS-6) and the Social Phobia Scale (SPS-6). Standard errors are shown

The ANOVA was significant for both the SIAS-6 (F(3,739) = 26.4,*p* < .001,η_p_^2^ = .10) and SPS-6 (F(3,739) = 25.3,p < .001,η_p_^2^ = .09) subscales. In both cases, this meant that people with neither fainting nor avoidance history scored the lowest on the scales, individuals with fainting history scored the second-lowest, and individuals reporting avoidance or both fainting and avoidance history scored the highest values. See Fig. 3 for the group differences. Mean scores and 95% confidence intervals are presented in Table 5.

**Table 5:** Differences on the Social Interaction Anxiety Scale (SIAS-6) and the Social Phobia Scale (SPS-6) for those with and without fainting and avoidance history on a subsample (*N* = 743)

SIAS				SPS		
		95% Confidence Interval				95% Confidence Interval
**Group**	**Mean**	**Lower**	**Upper**	**Mean**	**Lower**	**Upper**
Neither	3.47	2.81	4.13	3.05	2.37	3.73
Fainting	4.61	3.72	5.49	4.51	3.59	5.43
Avoidance	7.03	5.23	8.83	5.77	3.92	7.63
Both	7.12	6.59	7.65	6.84	6.29	7.39

The discrimination analysis regarding fainting history for the SIAS-6 showed that the overall Wilks’s Lambda was significant (Λ = .95, Χ^2^(1) = 40.03,*p* < .001) indicating that the SIAS-6 can discriminate between individuals with (M = -.32) and without a fainting history (M = .17). The model correctly classified 64.6% of the sample (κ = .23). Regarding avoidance history, the overall Wilks’s Lambda was also significant (Λ = .91, Χ^2^(1) = 71.24,*p* < .001) indicating that the SIAS-6 can discriminate between individuals with (M = -.33) and without avoidance history (M = .31). The model correctly classified 64.3% of the sample (κ = .30).

The same analysis regarding fainting history for the SPS-6 showed that the overall Wilks’s Lambda was significant (Λ = .94, Χ^2^(1) = 46.99,*p* < .001) revealing that SPS-6 can discriminate between individuals with (M = -.35) and without a fainting history (M = .19). The model correctly classified 64.6% of the sample (κ = .25). The overall Wilks’s Lambda was also significant (Λ = .92, Χ^2^(1) = 64.77,p < .001) regarding avoidance history, revealing that the SPS-6 can discriminate between individuals with (M = -.31) and without avoidance history (M = .29). The model correctly classified 63.8% of the sample (κ = .29).

### Clinical specificity

Regarding the group differences between the clinical and non-clinical samples, the results revealed that individuals with a diagnosis of social phobia (SIAS-6: M = 10.7, SD = 5.1; SPS-6: M = 10.3, SD = 5.9) scored higher on both the SIAS-6 (t(57) = 5.27, p < .001, Cohen’s d = 1.4) and SPS-6 (t(57) = 4.9, p < .001, Cohen’s d = 1.3) scales than members of the non-clinical control group (SIAS-6: M = 4.3, SD = 4.1; SPS-6: M = 3.8, SD = 4.1). The groups also differed on the bFNE scale (t(57) = 2.34, *p* = .02, Cohen’s d = .6) showing that individuals with social phobia related diagnosis (M = 23.5, SD = 7.9) scored higher than the members of the control group without a social phobia related diagnosis (M = 18.7, SD = 7.9). Mean scores and standard deviations are displayed in Table 2.

## Discussion

In the current study, our overarching goal was to test a translation of the SIAS-6 and SPS-6 scales. Further, we aimed to gain more information about the factor structure, reliability, validity, and clinical application potential of the SIAS-6 and SPS-6 questionnaires in both adults and adolescents. The latter group is particularly important as social anxiety often develops during adolescence [53], yet the psychometric properties of the SIAS-6 and SPS-6 are less researched in this age group compared to adults. Although SAD is one of the most frequent mental disorders with a lifetime prevalence of 5–13% [1–3], it often remains unrecognized [14]. Spontaneous recovery is rare [66] and those who receive treatment usually find help only 15–20 years after the onset of their symptoms [2]. SAD can have a fairly dramatic effect on the quality of life due to a higher level of disability in work, social life and leisure activities, and family life [67], severe adjustment problems, and a concurrent mood disorder [66, 68]. At the same time, it has been shown that only a 12-week course of cognitive-behavioral group therapy could significantly improve self-perceived quality of life in SAD patients [69]. Thus, quick yet effective screening, early identification, and treatment can reduce the negative consequences [42]. A previous systematic review [22] has shown that the SIAS-6 and SPS-6 are among the best measures for screening in primary care for the symptoms of SAD. However, previous studies call for further evidence on evidence on the psychometric properties and the applicability of the scales in various populations and cultures, as well as the elaborative validity of the short forms [22, 25, 32]. Our findings demonstrate that the SIAS-6 and SPS-6 have good psychometric properties and are reliable measures of social anxiety, assessing both anxieties regarding social interactions and fears related to the scrutiny of others in adults [20, 29, 30] and adolescents [22, 29, 41].

Across four subsamples, reliability, validity, and specificity analyses the SIAS-6 and SPS-6 were found to be internally consistent, with all items having moderate to high discriminant ability on the latent trait. This is in line with results found in different cultures, such as in Australian, American, Japanese, and South Korean samples [29, 49]. In correspondence with previous studies, the present study confirmed the two-factor structure of the SIAS-6 and SPS-6 scales [20, 25] both on adult and adolescent community samples. This result might be indicative of the validity of the “performance only” specifier of SAD in DSM-V, although the high correlation between the subscales, the similarity of the pattern of convergent validity for the scales, i.e., the lack of discrimination supports the result of recent meta-analyses, reviews, and empirical studies claiming that SAD is a latent continuum [23, 27, 28].

On the samples of adults and adolescents, the IRT analyses revealed that most of the items had very high discrimination ability indicating they are effective at discriminating individuals across a wide range of the latent trait. For the overall scores, in both cases, the analyses showed a positive shift on the latent trait. That is, the SIAS-6 and SPS-6 provide the most information (and least error) in the SD range of − 1 to 3 with a mean of about 1. This suggests that the scales could be appropriate for use in clinical practice.

Although the SIAS-6 and SPS-6 scales have satisfactory psychometric characteristics on an adolescent community sample, the IRT clearly showed that discrimination parameters are lower and the TIF curves were also flatter in adolescents compared to adults. This might be because social anxiety has a peak during the adolescent years [41–43]. Since the level of social anxiety is generally elevated in this population, the discrimination between typical or reasonable and atypical or excessive levels of social anxiety is much harder. This problem is also reflected in the TIF functions hinting at the possibility of lower reliability of the scales on an adolescent population (something that can also be seen in the McDonald’s ω values). Another explanation could be that social anxiety during adolescence is a more complex phenomenon with more possibly specifiers or subtypes than during adulthood. Nevertheless, since the most information is provided in the SD range of − 1 to 3, the results as a whole still indicate that the scales could be used to evaluate social anxiety in adolescents.

Our results regarding the convergent and divergent validity showed that both questionnaires correlated with measures of fear of negative evaluation, depression, and anxiety; whereas they did not correlate with a nonrelated construct of snake phobia. This is also in line with the findings of previous research using fear of negative and positive evaluation, depression, worry, and anxiety sensitivity measures [20, 25, 30].. Further, we also demonstrated that certain maladaptive cognitive emotion regulation strategies such as self-blame and rumination can facilitate social anxiety. Previous studies [36, 70–72] identified emotion regulation difficulties characterizing socially anxious individuals. For instance, rumination is frequently used by socially anxious individuals [70–72], as well as they have difficulties with the acceptance of emotional responses and controlling their impulsive reactions [40]. Further, socially anxious individuals are more prompt to blame themselves [40], probably because their attention is more self-focused [73]. From a clinical psychological point of view, the use of self-orientated affections and maladaptive emotion regulation strategies have a negative indication for mental health. This could also be relevant and effective in cognitive-behavioral interventions by, for instance, increasing levels of emotional clarity [74] or mindfulness training [75].

The correlational analyses, as expected, showed that both social anxiety scales correlated with measures of trait anxiety, depression symptoms, and fear of negative evaluation, while they did not correlate with a measure of another phobia (snake). Further, the linear regression analyses showed that maladaptive emotion regulation strategies (e.g. self-blame, rumination, and catastrophizing) were associated with social anxiety. This is in line with previous research about the role of emotion regulation in social anxiety and other anxiety disorders [37, 76]. The linear regression also revealed a slightly different pattern of emotion regulation strategies regarding social interaction anxiety and fears related to the scrutiny of others. Also, acceptance, which is an adaptive emotion regulation strategy positively predicted the SIAS-6 score. On the one hand, regarding social interaction fears, the items that measure the acceptance factor might rather assess resignation than acceptance. On the other hand, acceptance of the situation might not be helpful to cope with this type of fear as acceptance might facilitate avoidance. In contrast, positive reappraisal could help people to cope with such fears (see also [77]).

Regarding the potential of the clinical applicability of the scales, the IRT analyses showed that the items of SIAS-6 and SPS-6 can discriminate well between socially anxious and nonanxious respondents. This is in line with previous studies using samples of people with SAD or anxiety disorders [29, 30]. The scales can provide information over a relatively large proportion of the latent trait. The total scores are more sensitive to people with higher latent traits meaning that although the questionnaires can be used in a community sample, they would also be feasible in clinical settings [20, 29, 30]. The results of the discrimination analyses and the comparison of a clinical population with the community samples support this notion.

We found that scores on SIAS-6 and SPS-6 significantly discriminated those with a history of fainting from those without a fainting history and those with a history of social avoidance from those without a history of social avoidance. The results show that those with a history of social avoidance have the highest scores of all four groups as expected. This is in line with previous studies on blood, injury, and injection [78], animal phobias [79, 80]. The discriminant function analyses also revealed that scores on the two scales were capable of identifying those with a fainting history and those prone to avoidance. Therefore, the abbreviated six-item version of the SIAS-6 and SPS-6 scales may be particularly useful in an applied clinical setting where quick and efficient assessments are needed.

According to our results, the SIAS-6 and SPS-6 scales had good reliability on a clinical sample as well. Total scores showed negative correlations with age, which is in line with previous studies regarding other anxiety disorders [59, 79, 81, 82]. The group differences showed that people with a social phobia-related diagnosis scored higher on both the SIAS-6 and SPS-6 scales than those without a diagnosis. In sum, these findings were consistent with the previous studies in this paper, providing supportive evidence for the clinical utility of the SIAS-6 and SPS-6.

Furthermore, our results also endorse these measures as appropriate screening tools in primary care for the symptoms of SAD [22]. We have shown that the scales can also measure social anxiety in adolescents which is especially important in this context as social anxiety often develops during adolescence [42, 53, 66]. In the original paper describing the abbreviation of the SIAS and SPS [20], the authors recommend a cutoff score of 7 or higher for the SIAS-6 and 2 or higher on the SPS-6 scales. In contrast, in Study 4 and Study 5, the lower confidence interval for people reporting both avoidance and fainting/dizziness history was 6.6 for the SIAS-6 and 6.3 for the SPS-6 scale and was 8.9 for the SIAS-6 and 8.2 for the SPS-6 scale in the clinical sample. This suggests that while the originally proposed cutoff score of 7 or higher for the SIAS-6 could be used in Hungary, the cutoff score for SPS-6 should be 6 or higher instead of 2 or higher in Hungary. Nonetheless, further studies are needed to find the cutoff scores that could indicate to a physician to consider a further assessment of SAD.

Some limitations of the present study shall be noted. First and foremost, although we tested the factor structure, reliability, and several types of the validity of the SIAS-6 and SPS-6 scales, the cross-sectional nature is a shortcoming that precludes checking e.g., the treatment sensitivity of the measures. Second, although the large sample is a strength of our study, the gender imbalance may have confounded the results and could have made the comparison of females and males problematic. The female dominance in social anxiety is fairly well-described [83]. It is, therefore, important to verify these results in a more balanced sample. Further, the DSM-based questions used do not qualify as a clinical interview and do not necessarily mean that people endorsing the questions have SAD. Nevertheless, we could only assess a relatively small sample of diagnosed SAD patients, the two results are complementary and thus, are convincing that SIAS-6 and SPS-6 have a good discriminative ability.

Taken together, these limitations notwithstanding, the SIAS-6 and SPS-6 demonstrated adequate psychometric properties across adults, adolescents, and a clinical population. The questionnaires showed good construct, divergent, and predictive validity. Further, they were capable of discriminating individuals with a history of fainting and avoidance from people without such history, as well as people with SAD-related diagnoses from the members of a community sample. The scales are brief and easy to access, which could be important in a clinical setting or as part of a bigger survey. Shortness of the measures is also an important feature when the target population is old, has dementia or other cognitive impairments [17]. Previous studies [30, 31] demonstrated that both scales are sensitive to treatment. The predictive power of some maladaptive emotion regulation strategies on the SIAS-6 and SPS-6 scales provides a potential framework for cognitive-behavioral-based interventions in order to avoid the development of more severe psychopathological consequences [69, 74]. Therefore, the SIAS-6 and SPS-6 may be feasible tools to access social anxieties, including social interactions and fears related to the scrutiny of others in a wide variety of future research as well as screening, early identification, and monitoring of treatment efficiency in clinical practice.

## Supplementary Information

**Additional file 1: Supplementary Material 1.** The Hungarian version of the SIAS-6 and SPS-6 scales.

## Data Availability

The data that support the findings of this study are available from the corresponding author upon reasonable request.

## Footnotes

1 Please see the final Hungarian version, that we administered with all our participants, in Supplementary Material 1.

2 We also tested a one-factor model but that did not yield acceptable fit for adults (CFI = .886, TLI = .861, RMSEA = .115, 90%CI = [.111–.119], SRMR = .053) and for adolescents (CFI = .783, TLI = .735, RMSEA = .139, 90%CI = [.109–.170], SRMR = .081). Further, the AIC was smaller for the two-factor models (98,825 for adults and 2666 for adolescents) compared to the one-factor models (100,665 and 2641, respectively).
