# Supplementary material for: Psychometric properties of the social interaction anxiety scale and the social phobia scale in Hungarian adults and adolescents
Source: BMC Psychiatry. 2021 Mar 26;21:171. doi: 10.1186/s12888-021-03174-6 (PMC7995698; doi:10.1186/s12888-021-03174-6)
Supplement: Supplementary file 1 — Additional file 1: Supplementary Material 1. The Hungarian version of the SIAS-6 and SPS-6 scales. [file 12888_2021_3174_MOESM1_ESM.docx]

**Supplementary Material 1** – The Hungarian version of the SIAS-6 and SPS-6 scales along with the original statements in brackets. Items 1–6 are from the Social Interaction Anxiety Scale (SIAS), and items 7–12 are from the Social Phobia Scale (SPS).

|  |  | Egyáltalán nem | Egy kicsit | Vala-mennyire | Eléggé | Teljes mértékben |
| --- | --- | --- | --- | --- | --- | --- |
| 1 | Nehezemre esik másokkal felvenni a szemkontaktust. (*I have difficulty making eye contact with others.*) | 0 | 1 | 2 | 3 | 4 |
| 2 | Nehezemre esik kényelmesen elvegyülni a munkatársaimmal vagy évfolyamtársaimmal. (*I find it difficult mixing comfortably with the people I work with.*) | 0 | 1 | 2 | 3 | 4 |
| 3 | Feszült leszek, ha egy ismerőssel összefutok az utcán. (*I tense up if I meet an acquaintance on the street.*) | 0 | 1 | 2 | 3 | 4 |
| 4 | Feszültnek érzem magam, ha egyedül vagyok egy másik emberrel. (*I feel tense if I am alone with just one person.*) | 0 | 1 | 2 | 3 | 4 |
| 5 | Nehezemre esik másokkal beszélgetni. (*I have difficulty talking with other people.*) | 0 | 1 | 2 | 3 | 4 |
| 6 | Nehezemre esik ellentmondani mások véleményével. (*I find it difficult to disagree with another’s point of view.*) | 0 | 1 | 2 | 3 | 4 |
| 7 | Ideges leszek, hogy az emberek bámulnak, mikor az utcán sétálok. (*I get nervous that people are staring at me as I walk down the street.*) | 0 | 1 | 2 | 3 | 4 |
| 8 | Aggódom, hogy reszketni vagy remegni fogok, amikor mások figyelnek. (*I worry about shaking or trembling when I’m watched by other people.*) | 0 | 1 | 2 | 3 | 4 |
| 9 | Feszlült lennék, ha másokkal szemben kellene ülnöm a buszon vagy vonaton. (*I would get tense if I had to sit facing other people on a bus or train.*) | 0 | 1 | 2 | 3 | 4 |
| 10 | Aggódom, hogy olyat teszek, ami felhívja rám mások figyelmét. (*I worry I might do something to attract the attention of other people.*) | 0 | 1 | 2 | 3 | 4 |
| 11 | Egy liftben utazva feszült leszek, ha az emberek rám néznek. (*When in an elevator, I am tense if people look at me.*) | 0 | 1 | 2 | 3 | 4 |
| 12 | Feltűnőnek érzem magam sorban állás közben. (*I can feel conspicuous standing in a line.*) | 0 | 1 | 2 | 3 | 4 |
